# Cardiac remodeling and exercise tolerance in small for gestational age: a follow-up cohort study from preadolescence into adolescence

**DOI:** 10.3389/fcvm.2025.1654596

**Published:** 2025-11-18

**Authors:** Rommy H. Novoa, Dora Fabijanovic, Kilian Vellvé, Filip Loncaric, Mérida Rodríguez-López, Álvaro Sepúlveda-Martínez, Sebastian I. Sarvari, Brenda Valenzuela-Alcaraz, Francesca Crovetto, Rosa Faner, Alvar Agustí, Marta Sitges, Maja Cikes, Gabriel Bernardino, Isabel Blanco, Bart Bijnens, Fàtima Crispi

**Affiliations:** 1 BCNatal—Barcelona Center for Maternal-Fetal and Neonatal Medicine (Hospital Clínic and Hospital Sant Joan de Déu), Centre for Biomedical Research on Rare Diseases (CIBER-ER), Barcelona, Spain; 2 Universitat de Barcelona, Barcelona, Spain; 3 Centre for Biomedical Research on CardioVascular Diseases (CIBERCV), Institut Clínic Cardiovascular, Hospital Clínic, Barcelona, Spain; 4 Department of Cardiovascular Diseases, University of Zagreb School of Medicine, Zagreb, Croatia; 5 Department of Public Health and Community Medicine, Universidad Icesi, Cali, Colombia; 6 Fetal Medicine Unit, Department of Obstetrics and Gynecology, Hospital Clínico de la Universidad de Chile, Santiago de Chile, Chile; 7 ProCardio Center for Innovation, Department of Cardiology, Oslo University Hospital, Rikshospitalet, Oslo, Norway; 8 Department of Pulmonary Medicine, Respiratory Institute, Hospital Clínic Barcelona, Biomedical Research Networking Center on Respiratory Diseases (CIBERES), Barcelona, Spain; 9 FRCB-Institut D'Investigacions Biomèdiques August Pi i Sunyer (IDIBAPS), Barcelona, Spain; 10 BCN Medtech, Department of Information and Communication Technologies, Universitat Pompeu Fabra, Barcelona, Spain; 11 CREATIS, UMR 5220, U1294, F-69621, Univ Lyon, Université Claude Bernard Lyon 1, INSA-Lyon, CNRS, Inserm, Lyon, France; 12 Institució Catalana d'Investigació i Estudis Avançats (ICREA), Barcelona, Spain; 13 Katholieke Universiteit Leuven (KU Leuven), Leuven, Belgium

**Keywords:** small for gestational age, fetal cardiovascular programming, echocardiography, exercise tolerance, adolescence

## Abstract

**Background:**

Being born small for gestational age (SGA) affects 7%–10% of newborns and is associated with increased cardiovascular risk and reduced exercise capacity in adulthood with unclear underlying mechanisms. Cardiac remodeling and dysfunction occur in fetuses and children born SGA, but it is uncertain whether and how these changes persist into adolescence. The aim of the study was to assess resting cardiovascular morphology and function together with exercise tolerance in adolescents born SGA.

**Methods:**

A perinatal cohort of 30 adolescents born SGA (defined as birth weight below the 10th centile) and 28 normal birth weight controls in a tertiary university hospital in Spain was included. Participants were followed from preadolescence (age 7–12 years) into adolescence (age 12–17 years) with echocardiography and incremental cardiopulmonary exercise test (CPET).

**Results:**

Although signs of cardiac remodeling and dysfunction were evident in SGA preadolescents, no significant differences in left ventricular dimensions and deformation could be demonstrated in SGA adolescents. During the follow-up period, the SGA cohort had a significantly higher increase in left ventricular (LV) base-to-apex length (SGA mean 17.61 ± 6.78 vs. controls 13.44 ± 5.12; *p* = 0.011), resulting in different change of LV sphericity (−0.07 ± 0.11 vs. −0.17 ± 0.14; *p* = 0.010). Significant differences could be observed in SGA during exercise with reduced oxygen uptake **[**−0.07 L/min (−0.13 to −0.005); *p* = 0.035], expired carbon dioxide [−0.08 L/min (−0.15 to −0.01); *p* = 0.033], and peak expiratory flow rate [−0.11 L/s (−0.21 to −0.01); *p* = 0.029].

**Conclusion:**

Changes in cardiac shape and function, described in children born SGA, seem to be ameliorated in adolescence related to compensatory growth as compared to healthy controls. However, SGA adolescents had markedly reduced exercise tolerance.

## 1 Introduction

Cardiovascular disease (CVD) is a leading cause of death in adulthood (1), with 17.9 million annual global deaths (2). Subclinical CVD starts early in life, long before the clinical symptoms appear decades later, with strong evidence that it may start even before birth (3, 4). Robust epidemiological studies from the early 1990s established the prenatal influence on adult heart health (5). In particular, being born small for gestational age (SGA) has been recognized as a risk factor for CVD and mortality (6). Although the precise mechanisms underlying this association are not fully understood, prenatal cardiac changes are proposed as a main contributor. It is estimated that 7%–10% of newborns are born SGA (7), usually defined as birth weight or abdominal circumference below the 10th centile (8–10). A subgroup of SGA fetuses have abnormal fetoplacental Doppler [usually known as intrauterine growth restriction (IUGR)], with placental insufficiency being the most common cause (8–10). In this condition, the placenta delivers less oxygen and nutrients to the fetus and results in increased resistance and pressure overload to the fetal heart that needs to keep pumping blood to a stiffer and more resistant placenta (4). The fetus will adapt by remodeling the heart: SGA fetuses present larger, spherical, and hypertrophic ventricles with impaired relaxation (11, 12). Previous cohort studies suggest that prenatal cardiovascular changes observed in SGA will persist into childhood and preadolescence (13, 14). Recently, we showed that adults born SGA associate with subtle changes at rest in right ventricular geometry with preserved cardiac function. Interestingly, although the cardiac changes were less pronounced compared to children and preadolescents, adults born SGA had markedly reduced exercise capacity (15). Thus, cardiovascular remodeling associated with SGA seems to ameliorate from preadolescence into adulthood in baseline conditions but become evident during exercise. However, it is uncertain whether and how cardiovascular changes in SGA evolve from preadolescence into adolescence.

Our aim was to study the cardiovascular temporal evolution associated with SGA status. Thus, we planned a follow-up of a perinatal cohort from preadolescence into adolescence, including echocardiography and an exercise test.

## 2 Materials and methods

### 2.1 Study design

**Figure 1:**
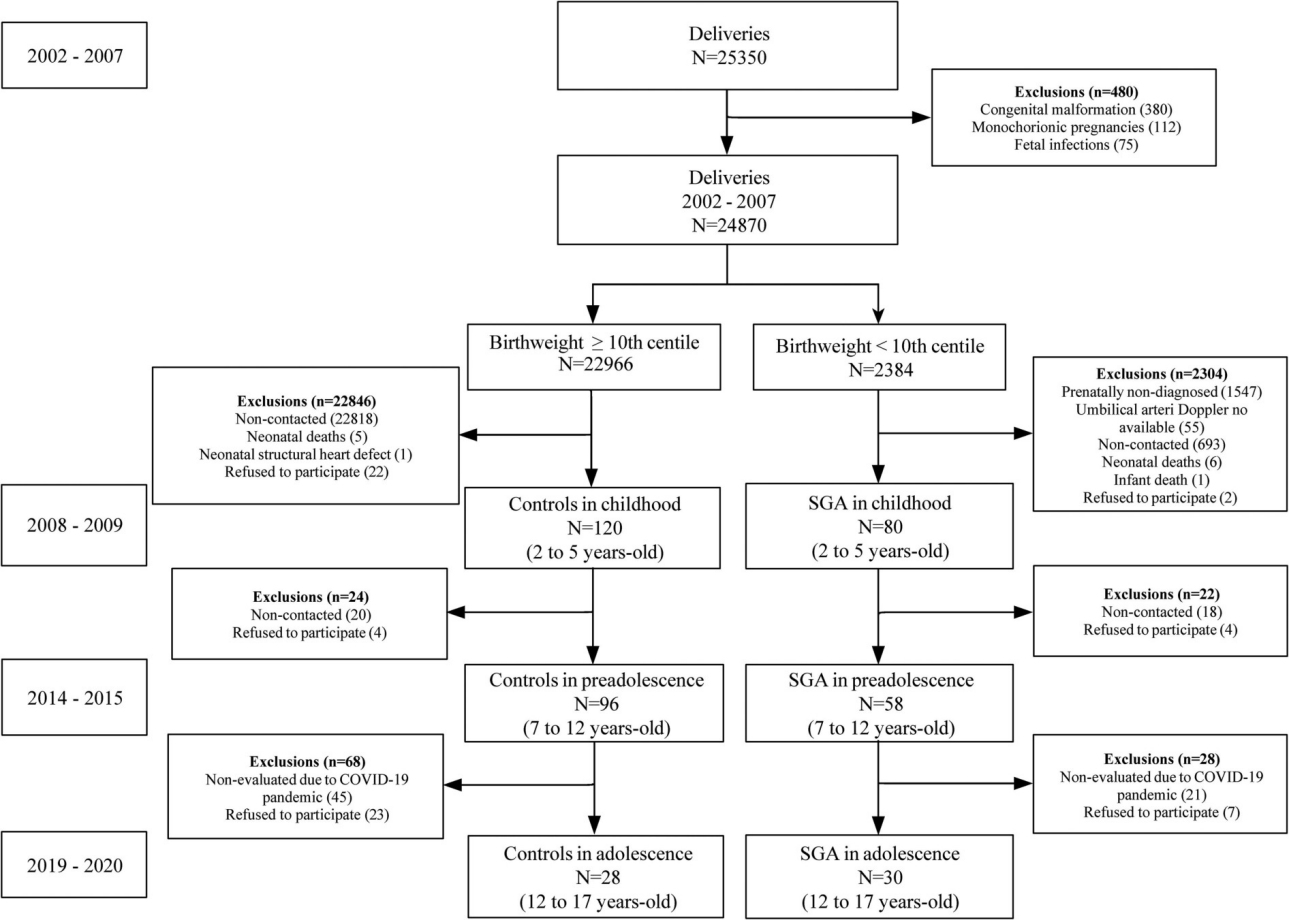
Flow diagram of the cohort from birth to adolescence. SGA, small for gestational age.

A prospective cohort study was conducted, including 30 participants born SGA and 28 controls with normal birth weight for gestational age, identified *in utero* and followed up from childhood (13), through preadolescence (14) and to adolescence. During follow-up, 68 controls and 28 SGA participants were lost, primarily due to the COVID-19 pandemic and refusal to continue with the study (Figure 1). SGA was defined as birth weight below the 10th centile, while the control group consisted of normally grown fetuses with a birth weight above the 10th centile (16). Exclusion criteria were neonatal congenital malformations or chromosomal defects, fetal infection, or multiple monochorionic pregnancies, current pregnancy or professional sport practice. Follow-up in preadolescence (age 7–12 years) included anthropometric measures, blood pressure measurement, and resting echocardiography. Anthropometric and echocardiographic results of the cohort at preadolescence age are detailed elsewhere (14). The study protocol in adolescence (age 12–17 years) included anthropometric measures, smoking and Physical Activity Questionnaire (PAQ-A), blood pressure measurement, resting echocardiography, and exercise test. The investigator responsible for performing and analyzing the measurements was blinded to the group allocation. The adolescent follow-up was conducted between October 2019 and March 2020 at the Hospital Clinic, a tertiary university hospital in Barcelona, Spain. The study protocol was approved by the Hospital Clinic Ethics Committee and written parental consent was obtained for all study participants.

### 2.2 Resting echocardiography

All preadolescent and adolescent participants underwent a (non-exercised) complete transthoracic echocardiogram at rest in left lateral decubitus position using Vivid iq (General Electric Healthcare) with a 2.5 MHz (M5S), simultaneously with electrocardiogram, following guidelines (17). Intraobserver reproducibility was not formally assessed. However, all examinations were performed by a single experienced operator (DF) using the same ultrasound equipment and a standardized, predefined protocol to minimize variability. The echocardiographic methodology has been previously validated and demonstrated high reproducibility in similar studies (18).

Offline analysis was performed with commercially available software (EchoPac version 203; General Electric Healthcare, Milwaukee, WI, USA). Cardiac dimensions were measured according to the recommendations of the American Society of Echocardiography and the European Association of Cardiovascular Imaging (18). Ventricular sphericity indexes were calculated as the ratio of the apex-to-base length and basal diameter, measured in apical four-chamber view. Relative wall thickness was calculated as the ratio of 2*posterior left ventricular (LV) wall thickness and the end-diastolic LV cavity diameter. Left and right atrial (LA, RA) areas were measured from the apical four-chamber view at maximum distension. Doppler recordings of the ventricular inflows and outflows were obtained. Systolic function was assessed by LV cardiac output from Doppler, and LV ejection fraction and right ventricular (RV) fractional area change by conventional two-dimensional (2D) echocardiogram. Mitral and tricuspid longitudinal ring motion was assessed using M-mode and real-time tissue Doppler from an apical four-chamber view. LV and RV strain were obtained from 2D speckle-tracking offline analysis (2D strain, EchoPac; General Electric Healthcare). When postsystolic shortening was present, the postsystolic index was calculated as the average of (peak postsystolic strain-peak systolic strain)/peak systolic strain*100 in 18 longitudinal LV segments. Diastolic function was assessed by E/A ratio, tissue Doppler early diastolic (e′) peak myocardial velocities, and isovolumic relaxation times.

To estimate the temporary evolution of the echocardiographic results, delta change was calculated by subtracting the value from adolescence minus that from preadolescence of each individual.

### 2.3 Exercise test

Adolescent participants completed a cardiopulmonary exercise test on a seated stationary electronically braked Ergoselect 100 (Ergoline) cycloergometer (range, 6–999 W) with all patients breathing room air. The equipment was calibrated before every test. Respiratory resting characteristics were obtained by forced spirometry and diffusion capacity for carbon monoxide (DLCO) (19, 20). Then, an incremental protocol, with a fixed cadence of 60 rpm, was applied, starting with 4 min of unloaded pedaling, 3 min of 25 W, followed by increments of 25 W every third minute until reaching 200 W or exhaustion. Respiratory gases were collected continuously using a breath-by-breath respiratory gas exchange system (Metalyzer 3B; Cortex Biophysik, Leipzig, Germany) including inspired and expired volumes and flow rates, oxygen consumption (V̇O_2_), and carbon dioxide production (V̇CO_2_). Blood pressure was measured at baseline and immediately after peak exercise, coinciding with the moment the patient discontinued the test due to symptom onset. The criteria for discontinuing the cardiopulmonary exercise test included muscle fatigue, severe dyspnea, chest pain, lightheadedness, and general physical discomfort.

### 2.4 Main outcomes and sample size calculation

The prespecified main outcomes were resting cardiac morphology and function by echocardiography, and cardiopulmonary response to incremental exercise testing. Sample sizes for the main outcome measures were calculated assuming an unknown but equal variance (previous studies suggest that variances among those born SGA and controls are similar), 80% power, 5% α error, and 1:1 allocation index. For echocardiography, a sample of 28 individuals per group was estimated to identify a 4% difference between groups in LV global longitudinal strain and assuming an SD of 1.16 and a standardized difference of 0.71 (14). For exercise testing, a sample of 17 individuals per group was estimated to detect a 33% difference in V̇O_2_ (15).

### 2.5 Statistical analysis

Stata/BE 18.0 (StataCorp LLC, College Station, TX, USA) was used for statistical analysis. Normality was tested using the Shapiro–Wilk test. Study groups were described using mean ± standard deviation (SD) values, median (interquartile range [IQR]) values, or frequencies and were compared using the Student's *t*-test, Wilcoxon–Mann–Whitney test, χ^2^ test, or Fisher’s exact test as appropriate. All reported *p*-values are two-sided, with *p* < 0.05 being considered statistically significant. Comparisons of cardiac parameters between SGA and controls in preadolescent age, adolescence, and the delta change between both periods was analyzed using linear or logistic regression analyses adjusted for sex, age, and body surface area at evaluation. To analyze group differences regarding exercise test variables, linear mixed models (LMMs) for repeated measures were fitted. Each point represented a certain load starting from the baseline. To study the load-dependence effect of the cardiopulmonary parameters during exercise testing, we used a longitudinal linear model including the load level, and adjusted for sex, age, and body surface area on the pooled temporal samples for each exercise testing group. CorelDraw version 24.3.0.571 was used for graphic design.

## 3 Results

### 3.1 Perinatal and current characteristics

The final number of participants included in the analyses was 30 adolescents born SGA [14 girls (47%)] and 28 control participants [14 girls (50%)]. All participants were of self-reported White ethnicity except for one African American SGA adolescent. All participants underwent echocardiography (at preadolescent and adolescent age) and the exercise test (in adolescence). The mean duration of follow-up of the cohort from preadolescence to adolescence was 5 years. Perinatal and current characteristics of the study population are displayed in Supplementary Table S1. Maternal characteristics were similar in both groups. By study design, SGA had lower birth weight and birth weight percentile with higher umbilical artery pulsatility index and similar gestational age at delivery as compared to controls. In adolescence, both groups had similar age and anthropometry. All participants were asymptomatic, and none of them received any specific cardiovascular treatment. Both groups presented similar rates of tobacco smoking and physical activity.

### 3.2 Resting cardiac morphology and function from preadolescence into adolescence

At rest, cardiac morphology and function of the study population from preadolescence into adolescence are shown in Table 1 and Supplementary Table S2. As previously reported, SGA preadolescents showed more spherical LV (mean ± SD, 1.90 ± 0.20 vs. 2.05 ± 0.19), higher relative wall thickness (0.36 ± 0.06 vs. 0.33 ± 0.04), reduced longitudinal strain (LV −21.50% ± 1.34 vs. −22.55% ± 1.30; RV −26.54% ± 3.18 vs. −29.43% ± 3.64%), higher postsystolic index (1.52 ± 0.75 vs. 0.84 ± 0.54), LV isovolumic relaxation time (−53.86 ± 12.56 ms vs. 45.75 ± 13.56 ms), and mitral E/A ratio (2.13 ± 0.34 vs. 1.78 ± 0.43) compared to controls.

**Table 1:** Resting echocardiographic results of the study population from preadolescence into adolescence.

Variable	Controls (*n* = 28)			SGA (*n* = 30)		
	Preadolescence	Adolescence	Delta change	Preadolescence	Adolescence	Delta change
Left ventricular morphometry						
Base-to-apex length (mm)	67.85 ± 5.24	80.75 ± 6.22	**13.44** **±** **5.12**	64.16 ± 6.86	81.62 ± 7.67	**17.61** **±** 6.78*
Basal diameter (mm)	33.25 ± 2.87	45.85 ± 3.44	13.16 ± 4.63	33.66 ± 4.53	45.42 ± 3.90	11.83 ± 5.13
Sphericity index	**2.05** **±** **0.19**	1.77 ± 0.13	**−0.17** **±** **0.14**	**1.90** **±** 0.20*	1.80 ± 0.14	**−0.07** **±** 0.11*
Relative wall thickness	**0.33** **±** **0.04**	0.36 ± 0.03	**0.03** **±** **0.05**	**0.36** **±** 0.06*	0.35 ± 0.03	**−0.01** **±** 0.05*
Right ventricular morphometry						
Base-to-apex length (mm)	63.42 ± 6.52	78.52 ± 8.57	16.30 ± 7.89	60.97 ± 6.93	79.45 ± 9.33	17.96 ± 8.4
Basal diameter (mm)	30.16 ± 4.31	34.02 ± 4.88	3.58 ± 5.46	30.35 ± 3.97	33.98 ± 4.63	3.36 ± 5.78
Sphericity index	2.13 ± 0.28	2.35 ± 0.39	0.27 ± 0.48	2.03 ± 0.28	2.36 ± 0.30	0.33 ± 0.47
Atrial morphometry						
Left atrial area (cm^2^)	10.87 ± 2.10	13.29 (10.41 ± 15.9)	2.84 ± 3.97	10.19 ± 2.50	12.82 (11.23 ± 17.4)	4.24 ± 3.43
Right atrial area (cm^2^)	9.35 ± 1.69	12.34 ± 3.0	2.87 (1.15–4.08)	9.10 ± 2.12	11.60 ± 2.96	2.21 (0.20–3.71)
Systolic function						
Heart rate (bpm)	85.79 ± 10.27	77.31 (68.75–84.91)	−7.04 ± 11.79	82.75 ± 9.20	78.89 (68.61–85.89)	−1.96 ± 15.31
LV cardiac output (L/min)	4.58 ± 0.89	5.66 (4.52–6.40)	1.17 ± 1.45	4.01 ± 1.27	4.91 (4.31–5.87)	1.24 ± 0.95
LV ejection fraction (%)	57.8 (56.30–60.58)	64.33 (62.56–66.11)	10.24 ± 7.66	58.63 (56.92–61.55)	64.16 (62.38–65.85)	8.23 ± 7.91
LV outflow peak velocity (m/s)	**1.02** **±** **0.11**	1.04 ± 0.17	0.04 ± 0.12	**1.10** **±** 0.15*	1.09 ± 0.15	0.03 ± 0.16
RV outflow peak velocity (m/s)	0.73 ± 0.13	**0.81** **±** **0.13**	0.08 ± 0.15	0.77 ± 0.16	**0.88** **±** 0.12*	0.07 ± 0.19
Pulmonary artery acceleration time (ms)	155.24 ± 28.38	**120.92** **±** **12.71**	−21.37 ± 25.62	156.46 ± 24.41	**130.03** **±** 13.88*	−20.90 ± 33.11
RV fractional area change (%)	43.31 ± 6.09	46.31 ± 7.40	1.35 (−6.38–5.6)	43.73 ± 5.47	45.67 ± 5.28	0.64 (−4.58–3.88)
Tricuspid ring displacement (mm)	23.12 ± 2.34	20.71 ± 1.80	−2.33 ± 2.59	22.86 ± 3.08	20.07 ± 1.31	−2.75 ± 2.90
Mitral s’ (m/s)	0.10 ± 0.02	0.13 ± 0.03	0.03 ± 0.03	0.10 ± 0.02	0.12 ± 0.03	0.02 ± 0.03
Tricuspid s' (m/s)	0.13 ± 0.02	0.12 (0.11–0.13)	−0.01 (−0.02–0.00)	0.13 ± 0.02	0.12 (0.11–0.13)	0.00 (−0.02–0.01)
LV global longitudinal strain (%)	**−22.55** **±** **1.30**	−20.19 ± 1.81	−2.60 ± 1.94	**−21.50** **±** 1.34*	−20.01 ± 2.06	−1.61 ± 2.51
RV global longitudinal strain (%)	**−29.43** **±** **3.64**	−24.25 ± 3.47	**−5.69** **±** **5.26**	**−26.54** **±** 3.18*	−25.38 ± 3.48	**−0.62** **±** 4.72*
Presence of postsystolic shortening (%)	6 (25.00)	8 (28.57)	1 (3.57)	11 (40.74)	13 (43.33)	2 (2.59)
Postsystolic index	**0.84** **±** **0.54**	5.83 ± 3.62	5.28 ± 3.54	**1.52** **±** 0.75*	5.00 ± 2.66	3.34 ± 2.77
Diastolic function						
Mitral E/A ratio	**1.78** **±** **0.43**	**1.77** **±** **0.46**	−0.05 ± 0.51	**2.13** **±** 0.34*	**1.91** **±** 0.38*	−0.21 ± 0.41
LV isovolumic relaxation time (ms)	**45.75** **±** **13.56**	69 (62–73.5)	20.83 ± 17.62	**53.86** **±** 12.56*	72 (65–73)	15.39 ± 11.58
Tricuspid E/A ratio^a^	1.94 ± 0.68	2.16 (1.62–2.56)	0.15 ± 1.09	2.12 ± 0.5	2.03 (1.74–2.42)	0.01 ± 0.80
Mitral e’ (m/s)	0.21 ± 0.03	0.17 ± 0.02	−0.03 ± 0.03	0.20 ± 0.03	0.18 ± 0.28	−0.02 ± 0.03
Tricuspid e’ (m/s)	0.17 ± 0.04	0.15 ± 0.03	−0.02 ± 0.04	0.16 ± 0.02	0.15 ± 0.03	−0.02 ± 0.04

Data expressed as mean ± SD, median (interquartile range), or *n* (%). SGA, small for gestational age; A, atrial inflow; E, early diastolic inflow; e’, myocardial peak velocity in early diastole; s’, myocardial peak velocity in systole; LV, left ventricular; RV, right ventricular; LA, left atrial; RA, right atrial. Delta change was calculated by subtracting the value from adolescence minus that from preadolescence. *P < 0.05 as compared to controls at similar age, calculated by linear or logistic regression adjusted for age, sex, and body surface area. Statistically significantly different (*p* < 0.05) variables are highlighted using bold text.

a Data available for 15 controls and 21 SGA preadolescents.

**Figure 2:**
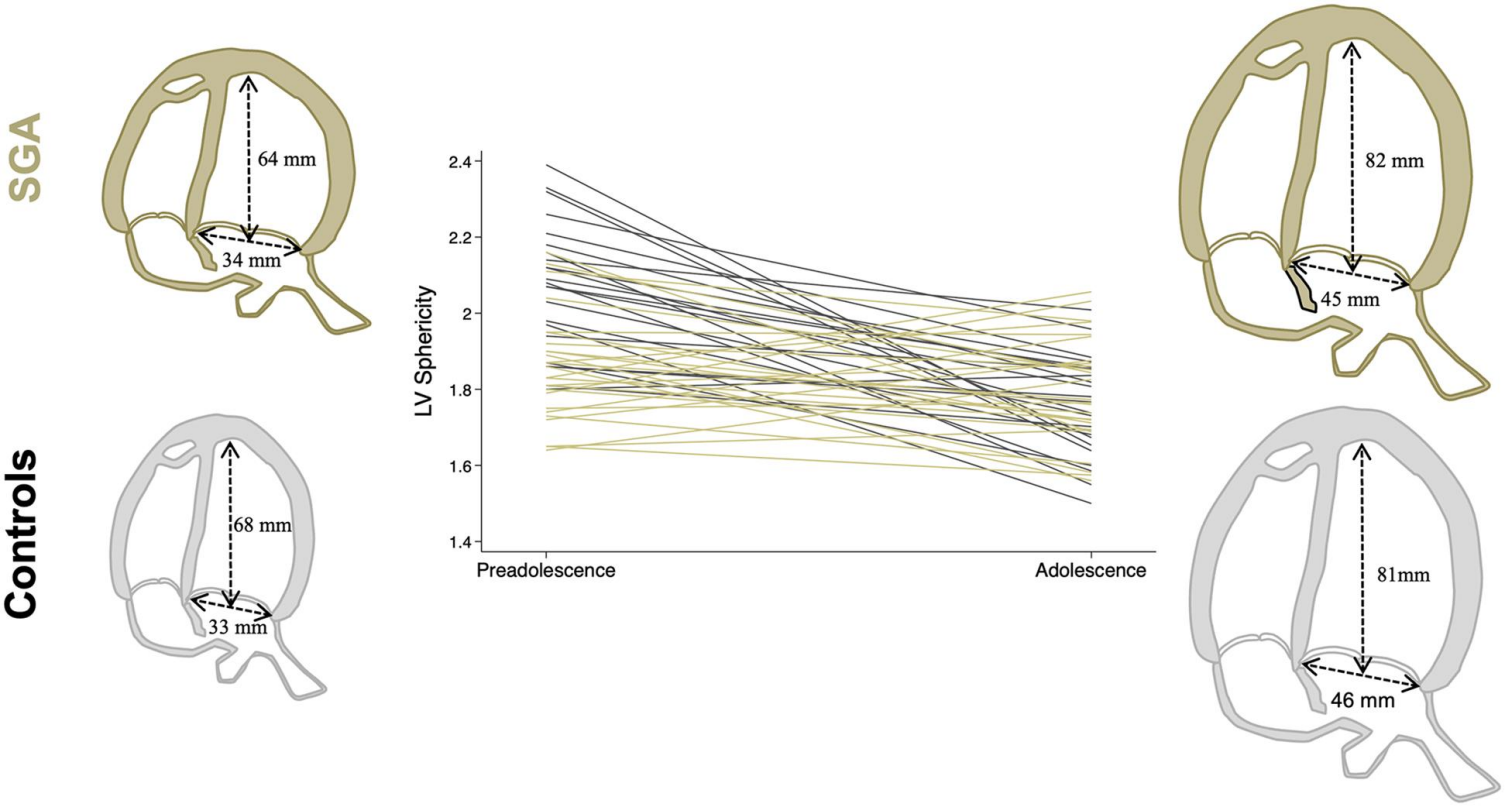
Individual change in LV sphericity during the follow-up period among the study groups (controls in gray and SGA in khaki). Illustrative cardiac figures, including mean average LV length and basal diameter in each study group. LV, left ventricular; SGA, small for gestational age.

During the follow-up period, the SGA cohort had a significantly higher increase in LV base-to-apex length (mean delta change 17.61 ± 6.78 vs. 13.44 ± 5.12), but equal increase of the LV basal diameter (mean delta change 11.83 ± 5.13 vs. 13.16 ± 4.36), resulting in a difference in the change of LV sphericity (mean delta change −0.07 ± 0.11 vs. −0.17 ± 0.14) between the groups (Figure 2). The mean change in RV global longitudinal strain was also smaller in the SGA cohort (−0.62 ± 4.72 vs. −5.69 ± 5.26). Overall, most echocardiographic parameters were similar in both study groups in adolescent age, except for increased mitral E/A ratio (mean 1.91 ± 0.38 vs. 1.77 ± 0.46) in SGA adolescents compared to controls.

### 3.3 Exercise tolerance

**Figure 3:**
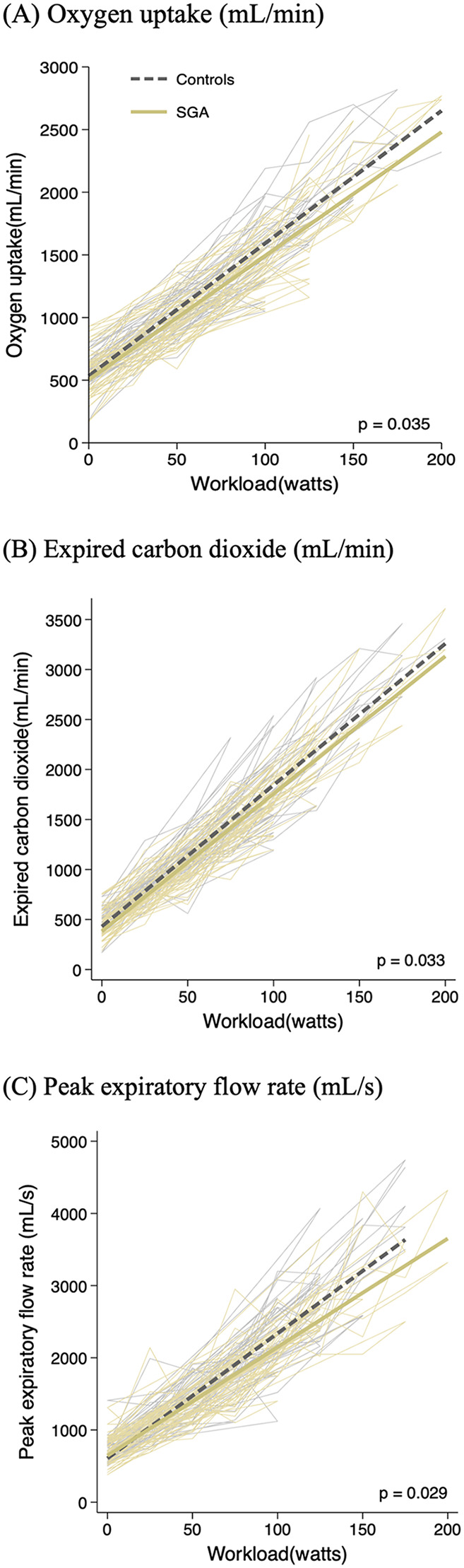
Individual and average curves of oxygen uptake **(A)**, expired carbon dioxide **(B)**, and peak expiratory flow rate **(C)** plotted according workload during exercise in controls (gray lines) and SGA (khaki lines) adolescents. *p*-value for estimated overall difference at each level of exercise in SGA compared to controls using multilevel mixed-effects linear regression adjusted for age, sex, and body surface area. SGA, small for gestational age.

An exercise stress test was performed in all adolescent participants. Respiratory baseline characteristics were similar between groups (Supplementary Table S3). The estimated overall difference between groups during exercise is shown in Table 2. Individual traces of the respiratory results as a function of the workload curves (respiratory results according to each workload slope) in SGA individuals and controls with 95% confidence intervals are shown in Figure 3. Significant differences were observed in SGA adolescents during exercise with reduced oxygen uptake [−70 mL/min (−130 to −5 mL/min)], expired CO_2_ [−80 mL/min (−150 to −10 mL/min)], and peak expiratory flow rate [−110 mL/s (−210 to −10 mL/s)] compared to controls.

**Table 2:** Estimated overall difference at each level of exercise between controls (*n* = 28) and small for gestational age (*n* = 30) adolescents.

Variable	Overall difference for exercise curves by workload in Watts	95% confidence interval	*p*-Value*
Ventilation (mL/min)	−1,880	−4,480 to 720	0.156
Inspired volume (mL/min)	−1,610	−4,070 to 850	0.200
Oxygen uptake (mL/min)	**−70**	**−130 to −5**	**0**.**035**
Expired carbon dioxide (mL/min)	**−80**	**−150 to** −**10**	**0**.**033**
Peak inspiratory flow rate (L/min)	−3.6	−9.6 to 2.4	0.236
Peak expiratory flow rate (L/min)	**−6** **.** **6**	**−12.6** to **−0.6**	**0**.**029**

* *p*-value comparing SGA versus controls, adjusted for age, sex and body surface area.

Bold indicate statistically significant results.

Although baseline blood pressure was similar in the study groups, SGA adolescents had higher systolic and mean blood pressure values at the end of the exercise test (Table 3). Systolic blood pressure at the final exercise significantly correlated with LV mass in the overall population, with a significantly different regression line in SGA adolescents who had significantly higher systolic blood pressure at final exercise per unit of LV mass as compared to than controls (*ρ* = 16.6; *p* = 0.027 when adjusted by age, sex, and body surface area) (Supplementary Figure S1).

**Table 3:** Blood pressure results at baseline and final exercise in the adolescent population.

Variable	Moment of exercise	Controls (*n* = 28)	SGA (*n* = 30)	Adjusted *p*-value*
Average workload achieved (Watts)		111 ± 37	107 ± 35	0.671
Systolic blood pressure (mm Hg)	Baseline	110.71 ± 10.32	114.25 ± 14.60	0.254
	Final exercise	**147.18** ± **21.31**	**159.19** ± **29.03**	**0**.**028**
Diastolic blood pressure (mm Hg)	Baseline	67.98 ± 6.26	68.52 ± 8.39	0.909
	Final exercise	73.16 ± 12.99	74.49 ± 9.49	0.475
Mean blood pressure (mm Hg)	Baseline	83.41 ± 7.17	83.98 ± 9.46	0.734
	Final exercise	**97.83** ± **11.67**	**102.73** ± **12.40**	**0**.**043**

Data expressed as mean ± SD.

* *p*-Value comparing SGA vs. controls, adjusted for age, sex, and body surface area.

SGA, small for gestational age.

Bold indicate statistically significant results.

## 4 Discussion

This study suggests reduced exercise tolerance in adolescents born SGA, despite an amelioration of the cardiac remodeling observed in younger ages.

### 4.1 Attenuated cardiac remodeling in SGA adolescents

In the present study, SGA-born adolescents showed improved cardiac remodeling compared to SGA at younger ages. *In utero*, SGA fetuses develop spherical, hypertrophic, and less efficient hearts in response to hypoxia, undernutrition, and pressure overload related to placental insufficiency (13, 21). This cardiac remodeling associated with SGA persists in childhood (13, 22, 23) and preadolescence (14, 24). In our study, which followed participants from preadolescence into adolescence, the ventricles of those born SGA showed a significantly greater increase in longitudinal length compared with controls. This was accompanied by a proportional increase in basal diameter, maintaining ventricular sphericity and resulting in a similar overall cardiac shape and postsystolic index between the SGA and control groups. Similarly, SGA preadolescents showed a thicker myocardial wall that was balanced into adolescence with a reduction in relative wall thickness. Regarding cardiac function, SGA adolescents improved longitudinal motion and myocardial deformation with similar changes in cardiac diastolic function. The persistence of an elevated E/A ratio in SGA preadolescents and adolescents may reflect impaired ventricular relaxation. Rather than indicating improved diastolic function, this pattern may suggest a predisposition to reduced ventricular compliance. These findings align with previous research showing subtle changes in cardiac morphology and function among SGA adolescents and young adults (15, 25), although these differences appear less pronounced than those observed at younger ages (11, 13, 14, 22–24, 26–30). This also corresponds with experimental data suggesting “catch-up” growth and cardiopulmonary structure and function during early adulthood in growth-restricted rats (31).

We hypothesized that the heart's catch-up growth and shape change in SGA adolescents may represent a compensatory response to hormonal, somatic, and pubertal loading changes (i.e., changes in height, weight, and blood pressure were also attenuated in SGA adolescents). This may occur within a broader biological disposition to resume growth along a genetically determined trajectory (32). Accelerated pubertal development has been reported in SGA adolescents, including greater visceral adiposity, reduced insulin sensitivity, and elevated insulin-like growth factor 1 concentrations (33, 34)—the latter being linked to enhanced cardiac growth and superior cardiac performance in young mice (35, 36). In addition, several studies indicate that cardiovascular risk factors in adolescence and early adulthood become less dependent on intrauterine hemodynamics and are more strongly influenced by lifestyle-related factors (37–39).

### 4.2 Reduced exercise tolerance

The present study suggests lower exercise tolerance in adolescents born SGA, with reduced oxygen consumption, expired carbon dioxide, and peak expiratory flow rate at each exercise level compared with participants of normal birth weight. To our knowledge, this is the first study to report the cardiopulmonary response of SGA adolescents during incremental exercise testing. Our data align with previous studies showing reduced exercise capacity in young adults born with low birth weight (15, 40, 41). We have recently demonstrated similar findings in a large cohort of young adults born SGA (15). Harris et al. reported that young adults born very prematurely and growth-restricted had lower mean lung function and exercise capacity than those born prematurely but with appropriate birth weight (40). Yang et al. also found reduced exercise capacity in adults with very low birth weight compared with term-born controls, independent of prematurity or bronchopulmonary dysplasia (41). The poorer exercise test results observed here are consistent with epidemiological evidence demonstrating a higher cardiovascular risk in adults born SGA (5, 42–44). We hypothesize that exercise testing acts as physiological stressor, revealing a suboptimal cardiorespiratory performance in this population. We also observed higher systolic blood pressure at peak exercise, which may be explained by reduced arterial distensibility (45, 46) and/or an exaggerated sympathetic tone response during exercise (47). However, this remains speculative, as arterial stiffness and sympathetic tone were not directly assessed. It is possible that SGA adolescents are unable to increase stroke volume effectively—through deformation and/or heart rate changes—potentially due to right heart limitations, as previously suggested in our adult study (15). This would indicate that overall cardiovascular performance during exercise is compromised in SGA adolescents.

These findings are consistent with animal studies suggesting an impaired cardiac response to stress in SGA (47). Overall, the data support the idea that reduced exercise tolerance may reflect underlying cardiovascular and pulmonary changes associated with SGA and warrant further investigation. This speculation is further strengthened by our data indicating that adolescents born SGA exhibited significantly higher systolic blood pressure at peak exercise for the same LV mass as controls, suggesting an overall cardiopulmonary maladaptation to exercise.

### 4.3 Strengths and limitations

This study has some strengths and limitations. The main strength lies in the well-characterized cohort of SGA participants identified during fetal life. This allowed for accurate classification of SGA fetuses (48) and controls through prenatal ultrasonography, along with precise records of birth weight and perinatal complications. We also conducted a comprehensive echocardiographic evaluation at rest, and for the first time, applied an exercise test to assess the cardiopulmonary tolerance of SGA adolescents. Despite the relatively small sample size, it met the target determined by our sample size calculation.

We acknowledge several limitations in our study. Although results were adjusted for key potential confounders such as sex, age, and body surface area, the study was not designed to evaluate the influence of additional factors (e.g., anemia or other social determinants). For example, because our study population was predominantly White, the generalizability of the findings to other ethnic groups—such as African Americans—may be limited. Similarly, pubertal development may have influenced the results; however, data on this variable were unavailable and could not be included in the analysis. The relatively low sample size also precluded a subanalysis comparing SGA and IUGR cases. Although the cardiopulmonary exercise test has certain technical limitations—such as potential submaximal performance, measurement variability, and the lack of universally standardized reference values—it remains a valuable physiological tool for assessing exercise intolerance and dyspnea, contributing to clinicians' understanding and diagnostic decision-making. The limited availability of high-quality normative data for this age range constrains the establishment of robust reference standards. Finally, the observed changes are subclinical, with most measurements remaining within expected normal ranges. Whether these findings persist long term or translate into a higher risk of cardiac disease remains to be determined.

### 4.4 Clinical and research relevance of the findings

The relationship between increased incidence of cardiovascular disease in adulthood and growth restriction *in utero* have been reported in previous studies (5, 42–44). However, the specific mechanisms remain incompletely understood. Fetal cardiac remodeling related to pressure/volume overload secondary to placental insufficiency has been consistently shown to persist into childhood (4, 11, 13, 22, 23). Interestingly, although cardiac remodeling appears to attenuate over time, this adaptation is insufficient to fully compensate for the loss of functional capacity. Exercise testing may therefore unmask the increased CVD susceptibility observed in individuals born SGA at older ages.

Since SGA affects 7%–10% of the population, well-established preventive strategies targeting this higher-risk subgroup could improve quality of life and reduce morbidity and mortality (4, 49). Future research is warranted to further understand the long-term cardiovascular changes in this population and to identify effective preventive measures from the earliest stages of life.

## 5 Conclusions

In conclusion, adolescents born SGA demonstrate reduced exercise tolerance despite the absence of major structural cardiac abnormalities. However, these differences in exercise performance appear to be subtle and subclinical. Future studies are warranted to better understand long-term cardiovascular implications of SGA and to identify potential preventive measures for this at-risk population.

## Data Availability

The raw data supporting the conclusions of this article will be made available by the authors, without undue reservation.
