# Supplementary material for: Cardiac remodeling and exercise tolerance in small for gestational age: a follow-up cohort study from preadolescence into adolescence
Source: Front Cardiovasc Med. 2025 Nov 18;12:1654596. doi: 10.3389/fcvm.2025.1654596 (PMC12669170; doi:10.3389/fcvm.2025.1654596)
Supplement: Supplementary file 1 [file Datasheet1.pdf]

# *Supplementary Material*

**Supplementary Table 1.** Perinatal and current characteristics of the study population

| | **Controls (n=28)** | **SGA (n= 30)** | ***P* value** |
|---|---|---|---|
| Maternal characteristics | | | |
| Maternal age, years | 42.88 ± 5.43 | 42.82 ± 4.41 | 0.969 |
| Low socioeconomical status | 0 (0.00) | 4 (13.30) | 0.110 |
| Smoking[†] | 3 (12.50) | 9 (32.14) | 0.113 |
| Prenatal fetoplacental ultrasound | | | |
| Umbilical artery pulsatility index, Z score | **−0.145 (−0.47 to 0.68)** | **3.93 (0.98 to 5.49)** | **0.030** |
| Middle cerebral artery pulsatility index, Z score | -0.23 ± 1.1 | -1.32 ± 1.70 | 0.163 |
| Pregnancy complications | | | |
| Preeclampsia | 0 (0.00) | 3 (10.71) | 0.240 |
| Gestational diabetes | 1 (4.17) | 3 (12.00) | 0.609 |
| Use of steroids in pregnancy | 6 (25.00) | 13 (52.00) | 0.079 |
| Perinatal data | | | |
| Female sex | 14 (50.0) | 14 (46.67) | 0.508 |
| Birthweight, g | **3108.13 ± 697.90** | **1899.4 ± 737.84** | **< 0.001** |
| Birthweight percentile | **73 (38.5 – 95)** | **1 (0 – 2)** | **< 0.001** |
| Gestational age at delivery, weeks | 38 (34.3 - 40) | 36.75 (32.75 – 39.5) | 0.297 |
| Preterm delivery[Ψ] | 10 (40.00) | 14 (50.00) | 0.583 |
| Umbilical artery cord pH | 7.26 ± 0.95 | 7.23 ± 0.07 | 0.305 |
| Major neonatal morbidity | 2 (8.33) | 3 (12.00) | 1.000 |
| Offspring's characteristics at adolescence | | | |

| | | | |
|---|---|---|---|
| Age at evaluation, years | 16.27 (13.47–16.71) | 15.15 (13.29–16.02) | 0.093 |
| White ethnicity | 28 (100) | 29 (96.67) | 1.000 |
| Weight, kg | 58 (52–62.5) | 52 (48–68) | 0.691 |
| Height, cm | 163.82 ± 9.38 | 162.77 ± 9.31 | 0.518 |
| Body mass index, kg/m$^2$ | 20.70 (19.21–23.57) | 21.28 (19.40–23.15) | 0.864 |
| Body surface area, m$^2$ | 1.63 ± 0.19 | 1.62 ± 0.22 | 0.484 |
| Smoking[†] | 1 (3.57) | 1 (3.33) | 0.706 |
| PAQ-A-Physical activity Score[#] | 8.25 ± 1.05 | 10 ± 1.29 | 0.302 |

Data expressed as mean ± SD, median (interquartile range), or number (%).
SGA indicates Small for Gestational Age.
Right column shows *P* values for Student t test, Wilcoxon–Mann–Whitney test, and χ2 test appropriate.
[†]Smokers were those individuals who smoked on a regular basis (≥1-2 cig/day). Individuals who had stopped smoking at least 1 year before the study onset were labelled as non-smokers.
[Ψ]Preterm delivery defined as gestational age at delivery before 37 weeks.
[#] Physical activity score according to PAQ-A (Physical Activity Questionnaire for Adolescents).

**Supplementary Table 2.** Resting (non-exercised) echocardiographic results normalized by body size of the study population from preadolescence into adolescence.

| | Controls (n = 28) | | | SGA (n = 30) | | |
|---|---|---|---|---|---|---|
| | Preadolescence | Adolescence | *Delta change* | Preadolescence | Adolescence | *Delta change* |
| **Ventricular morphometry** | | | | | | |
| LV base-to-apex length, mm/m$^2$ | 56.56 ± 6.82 | 49.94 ± 4.55 | *-7.38 ± 6.04* | 55.25 ± 7.11 | 50.94 ± 4.61 | *-4.06 ± 6.97* |
| LV basal diameter, mm/m$^2$ | 26.65 (24.81; 30.38) | 28..39 ± 2.83 | *0.37 ± 3.32* | 28.13 (25.56 32.91) | 28.38 ± 2.66 | *-0.44 ± 4.26* |
| RV base-to-apex length, mm/m$^2$ | 52.32 ± 5.56 | 48.50 ± 5.5 | *-5.14 ± 5.95* | 52.42 ± 6.41 | 49.43 ± 5.6 | *-2.89 ± 6.61* |
| RV basal diameter, mm/m$^2$ | 24.96 ± 4.22 | 21.06 ± 3.3 | *-5.08 ± 5.14* | 26.15 ± 3.97 | 21.14 ± 2.46 | *-5.00 ± 4.46* |
| **Atrial morphometry** | | | | | | |
| LA area, cm$^2$/m$^2$ | 9.02 ± 1.66 | 7.98 (6.44 – 9.61) | *-0.90 ± 2.48* | 8.63 ± 1.59 | 8.64 (7.34 – 10.15) | *0.35 ± 2.17* |

| | | | | | | |
|---|---|---|---|---|---|---|
| RA area, $cm^2/m^2$ | 7.74 ± 1.23 | 7.58 ± 1.53 | *-0.13 (-1.96 – 0.35)* | 7.71 ± 1.22 | 7.12 ± 1.21 | *0.07 (-1.98 – 0.80)* |
| **Systolic function** | | | | | | |
| LV cardiac output, L/min/kg | 0.11 (0.10; 0.14) | 0.10 (0.08; 0.11) | *-0.03 (-0.04; 0.003)* | 0.11 (0.10; 0.12) | 0.09 (0.08; 0.11) | *-0.02 (-0.04; -0.007)* |
| Tricuspid ring displacement, $mm/m^2$ | 19.32 ± 3.06 | 12.87 ± 1.80 | *-6.69 ± 2.79* | 19.63 ± 2.60 | 20.07 ± 1.31 | *-2.92 ± 2.19* |

Data expressed as mean ± SD or median (interquartile range).
All cardiac morphometric parameters were normalized by dividing it by Body Surface Area with the exception of LV cardiac output normalized by weight in kg.
Delta change was calculated by subtracting the value from adolescence minus tat from preadolescence.
SGA indicates Small for Gestational Age; LV, left ventricular; RV, right ventricular; LA, left atrial; RA, right atrial
**P* < 0.05 compared to controls, calculated by linear or logistic regression adjusted for sex and age. None of the parameters showed statistical significant differences among the 2 study groups.

**Supplementary Table 3.** Resting (non-exercised) respiratory characteristics of the adolescent population

| | **Controls** (n=28) | **SGA** (n=30) | ***p-value*** |
|---|---|---|---|
| ***Forced spirometry*** | | | |
| $FEV_1$ (L) | 3.34 ± 0.85 | 3.15 ± 0.69 | 0.263 |
| $FEV_1$ (% pred.) | 96.39 ± 13.19 | 93.48 ± 10.84 | 0.340 |
| FVC (L) | 3.78 ± 0.87 | 3.69 ± 0.70 | 0.993 |
| FVC (% pred.) | 93.17 ± 11.35 | 92.06 ± 11.20 | 0.850 |
| $FEV_1$/FVC (%) | 88.16 ± 7.03 | 86.24 ± 7.37 | 0.178 |
| ***CO Diffusing capacity of the lung***$^{‡}$ | | | |
| DLCOsb (mL/min/mmHg) | 25.55 ± 6.40 | 24.06 ± 7.63 | 0.767 |
| DLCO (% pred.) | 84.26 ± 11.70 | 80.51 ± 12.84 | 0.397 |
| VA (L) | 4.59 ± 1.05 | 4.30 ± 1.05 | 0.344 |
| VA (% pred.) $^{‡}$ | 87.93 ± 10.72 | 86.03 ± 12.10 | 0.895 |
| KCO (DLCO/VA) | 5.59 ± 0.70 | 5.55 ± 0.61 | 0.976 |
| KCO (% pred.) | 98.02 ± 14.01 | 98.79 ± 8.31 | 0.604 |
| ***Inspired and expired gases**** | | | |
| $\dot{V}O_2$ (mL/min/kg) | 10.21 ± 2.67 | 9.72 ± 2.72 | *0.843* |
| $\dot{V}CO_2$ (mL/min/kg) | 8.76 ± 2.84 | 7.92 ± 2.24 | *0.321* |
| Respiratory Quotient | 1.19 ± 0.17 | 1.20 ± 0.14 | *0.767* |
| VE (L/min) | 15.59 (13.19 -17.74) | 14.35 (11.23 – 17.98) | *0.358* |
| VE/$\dot{V}CO_2$ | 31.29 (29.73 - 32.69) | 31.40 (29.37 – 33.66) | *0.529* |
| $O_2$ pulse (mL/beat/min) | 5.16 (4.56 – 6.61) | 5.25 (3.76 – 6.63) | *0.593* |

SGA denotes Small for Gestational Age; $FEV_1$, forced expiratory volume in the 1$^{st}$ second; FVC, forced vital capacity; DLCOsb, diffusing capacity of the lung for carbon monoxide single breath; VA, alveolar volume; KCO, transfer coefficient; $\dot{V}O_2$, oxygen consumption; $\dot{V}CO_2$, carbon dioxide production; VE, minute ventilation; VE/$\dot{V}CO_2$, ventilatory equivalent for $CO_2$ at anaerobic threshold.
Data shown as mean ± SD or median (IQR).
P-value adjusted for age, sex, body surface area. Significantly different ($p<0.05$) variables are highlighted using bold text.
$^{‡}$Data available for 17 controls and 16 SGA. * Data available for 20 controls and 17 SGA

**Supplementary Figure 1.** Regression line analyse between blood pressure at end of exercise and LV mass indexed

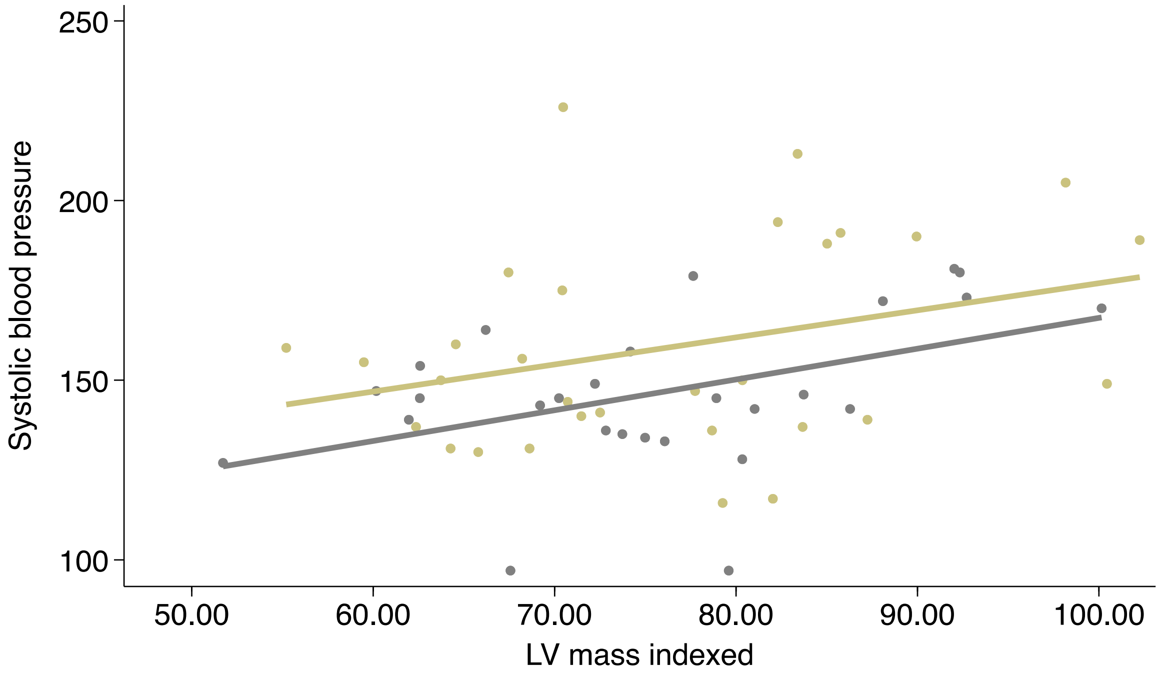


Regression line showing a positive association between the blood pressure at end of exercise and LV mass indexed (ρ = 16.6; P=0.065, and P = 0.027 when adjusted by age, sex, and body surface area). Adults born small for gestational age (SGA) present a significantly different regression line and have lower systolic blood pressure per unit of LV mass than controls.
